# Loneliness and social isolation among the older person in a Swiss secure institution: a qualitative study

**DOI:** 10.1186/s12877-022-02764-7

**Published:** 2022-02-01

**Authors:** Félix Pageau, Helene Seaward, Elmar Habermeyer, Bernice Elger, Tenzin Wangmo

**Affiliations:** 1 Institute for Biomedical Ethics, University Basel, Basel, Switzerland; 2 Psychiatrische Universitätsklinik, Klinik für Forensische Psychiatrie, Zurich, Switzerland; 3 Unit of Health Law and Humanitarian Medicine, Center for legal medicine, University of Geneva, Geneva, Switzerland

**Keywords:** Loneliness_1_, Social Capital_2_, Prisoners_3_, Trust_4_, Forensic psychiatry_5_, Older adult_6_

## Abstract

**Background:**

A pandemic of loneliness is hitting the aging population. As COVID19 forced us to isolate ourselves, we are in a better position to understand consequences of social distancing. The recent literature showed that older incarcerated adults are particularly at risk of health-related complications due to isolation in the prison environment, reducing their social capital. Mental and physical health can be severely affected by loneliness and social isolation, especially in prison.

**Methods:**

Our qualitative study investigates the view of older persons deprieved of their liberty on loneliness and social isolation pertaining to their mental health. We interviewed 57 older participants, including imprisoned individuals and forensic patients, following a semi-structured interview guide. During the data management and data analysis process, we excluded 7 interviews which were of poorer quality. Thereafter, we analyzed the remainders following a thematic approach.

**Results:**

Most interviewees experience loneliness following lack of significant human relationships in prison. Making friends appears to be a challenge for all the participants, because, for one thing, they do not find people with similar interests. Also, secure institution setting aggravates isolation due to the restrictions of movement placed such as rules concerning movement between floors, hindering intimate relationship, and separation between friends. Moreover, contact with prison personnel is limited and lack social capital (e.g. trust).

**Conclusion:**

To our knowledge, this study is one of the first to present incarcerated persons’ perspective on loneliness, social isolation and poor social capital in the Swiss prison setting. These has been reported to cause health problems both somatic and psychological. Our participants experience these deleterious factors in detention. As prisons have the possibility to become a health-promoting environment through connectedness, friendship, and trust promotion, stakeholders need to better their social capital.

**Supplementary Information:**

The online version contains supplementary material available at 10.1186/s12877-022-02764-7.

## Introduction

The former Prime Minister of UK, Theresa May, said that “Loneliness is one of the greatest public health challenges of our time” ([1], p. 1), as she launched the first cross-Government strategy to tackle this problem. Loneliness is the reduced number or quality of contacts with family and friends leading to unfulfilled intimate and social needs [2–4]. It has been linked to depression in multiple studies [5–9]. Poorer sleep, higher vascular resistance, and slower wound healing are adverse effects of loneliness for older adults’ health [10]. Furthermore, loneliness alone is an independent risk factor for mortality in old age [2, 11, 12]. Loneliness is also closely related to poor quality of life and mental health [7, 13, 14].

Social isolation, a notion related to loneliness, is the inadequacy and poor quantity of social relations with others [3, 4, 12, 15]. Both are linked to mental [5, 6, 9, 16, 17] and physical health problems [2, 4, 8, 15, 17, 18] such as depression and cardiovascular disease [10, 19]. Among older persons, both loneliness and social isolation have been related to a more rapid cognitive decline in a Spanish longitudinal study on aging [20]. These results are similar to those of a British study with data from the English Longitudinal Study of Ageing [21].

On the opposite end is the notion of social capital, which “refers to the socio-structural resources (e.g., emotional or instrumental support) that accrue through shared norms and values within durable relationships […]. Social capital may be cognitive (e.g., trust) or linked to network structure (e.g., network size and composition)” ([22], p. 25). This concept has been explored thoroughly and presented vastly in the recent literature [23]. As a well-studied concept, it has multiple definitions [23], but is linked to trust, social networking, reciprocity, bonds within a social network, social norms, participation, values, belonging, support, communication, social cohesion, solidarity, and safety [24]. The bonding social capital pertaining to family connectedness has shown to reduce mental health related social isolation [25].

Loneliness, social isolation, and lacking social capital are even more common in prisons, a harsh environment [26], where maintaining and forming meaningful relationships is challenging. Ami [26], (p.277) wrote: “some incarcerated individuals learn to be “invisible” and disconnected from others. They retreat into themselves, trust no one, and lead isolated lives of quiet desperation”. This leads to not entrusting others with personal health or intimate details. A strong relationship has been established in the recent literature between psychological distress and social isolation in prison [27]. Even though, detained individuals generally feel alone and abandoned, connectedness with the outside (family and friends) can reduce psychological harm and substance use [28]. However, maintaining relationship with family members and friends is also limited with prison rules regarding visitations [29–31]. In this paper we explore incarcerated older adults’ experience of social capital or how their social capital influenced their experience of loneliness. We aim to better understand their perspective on these issues to help reduce social isolation and loneliness in prison and forensic institution. This is also to say that social capital and connectedness are essential for physical and mental health.

## Materials and methods

### Study aim, design and setting

Scarcity of data pertaining to elders in prison is well-known [32, 33]. Research on mental health topics in prison is also lacking [34]. Therefore, our team elaborated an exploratory qualitative research to better understand the perspective of older individuals deprived of their liberty on their mental health. It was named *Agequake II* in relation to the increasing number of aging adults in prison, creating a wave as an earthquake. The aim of this study is to have a better understanding of the older persons in secure institution general experience on aging and mental health care. Data analysed and presented in this paper stems from this larger project.

We followed the reporting guidelines of “Journal article reporting guidelines” for qualitative research [35], which includes COREQ-32 guidelines [36].

A total of 15 institutions (prisons and forensic-psychiatric units) participated in our study and thereby supported participant recruitment. For qualitative data collection with older persons in prisons, the inclusion criteria were: (1) to be 50 years and older and (2) to have at least one contact with mental health services. As incarcerated person age more rapidly than their counterparts outside of prison, 50 year of age was decided as a cut-off point [37, 38]. The potential participant was excluded if his/her (1) mental health was too instable to participate in a study or (2) the administration did not allow him/her to participate (e.g. due to dangerousness or solitary confinement).

A contact person at the institution carried out the recruitment using purposive sampling. This person handed out study information, informed consent to prospective participants. The interview schedule was handed out by this person. On the day of the interview, confidentiality was assured and stated once more by the interviewer after informing the participant about the purpose of the study. Interviewee’s right to refusal to participate at any time during the whole process was stated again. Subsequently, written informed consent was obtained. There was no compensation provided for this study participation. French, German, Swiss-German or English was used to complete interviews. It was participant choice pertaining to the language used.

A total of 57 older incarcerated persons were interviewed for the project in total (1 interview per participant) between December 2017 to December 2018. Interviews were held in person in a separate location at the institution assuring privacy to speak freely. Two research assistants trained in qualitative interview techniques conducted the discussions. They were working on their doctoral degree at that time. Participants met with their interviewer on the day of the interview for the first time. No relationship existed prior to data collection between both parties.

Swiss prisons are similar to any Western prison, as for swiss forensic units, they are close to what other psychiatry forensic units in Canada and other developed countries are. In prison, people are treated as detainees under legal ruling, as for forensic unit they are considered as psychiatric patients. Measures are applied when imprisonment alone is judged insufficient to reduce the risk of recidivism. Forensic-psychiatric treatment and regular evaluations of its effect and of dangerousness are then mandatory for the offender. The Swiss Criminal Code (SCC) regulates this incarceration by means of various articles such as Article 64 SCC or 59 SCC. The preceding – therapeutic measure (Art. 59 SCC) - ensures treatment as an in-patient measure till successful in relation to the underlying mental illness associated with the crime. The person is then released when the therapeutic measure is medically considered successful. At the conclusion of a criminal trial and if mental health conditions cannot be treated and/or if the person convicted for a crime is judged dangerous, an indefinite imprisonment is pronounced following a security measure (Art. 64 SCC). This can be converted to Art. 59, if a person is considered treatable later. Thus, measure persons fall under any of those two (therapeutic or security measure). Noticeably, social interactions are very limited in prison but more permissible in forensic facilities, according to our results and what we know as prison experts.

The total number of study participants was decided based on the saturation principle [39, 40]. That is, we conducted data analysis while collecting the data, a process that allowed us to judge when no new information is obtained with new data collection [39, 40]. The interviews took 70 min on average (range from 16 to 120 min). 7 interviews were excluded during data analysis due to poorer quality. Hence, data from 50 older incarcerated persons’ interviews are included for this paper. Out of these 50 participants, 14 were from forensic institutions and 36 were from prisons. Nine older incarcerated persons had penal sentences with the remaining 41 serving a security or therapeutic measure. They therefore receive therapy (from the German phrase ‘therapie begleitung’ or an obligation to go on therapy). The 9 participants serving penal sentences were included because they have been in contact with mental healthcare at least once during their imprisonment. Although, they may or may not be receiving regular therapy, we found their perspective on isolation informative for our article.

By linguistic region, 19 participants were enrolled from the French-speaking part and 31 from German-speaking part of Switzerland. Mean age of interviewees was 61 years old, ranging between 50 and 76 years; and 8 out of 50 participants were women. The semi-structured interview guide is summarized in Table 1. We have also included the *Interview Guide for Imprisoned Adults* as a Supplementary file.

Interviews were transcribed verbatim in the language of the interview. Swiss German interviews were written in Standard German as it is common practice for Switzerland. The interviews were verified for the quality and accuracy of the transcriptions and translation. Identifying information were anonymized. Interview transcripts were not returned to the participants for verification as this process would mean that their data becomes known to prison personnel controlling in-coming mails.

### Data analysis

A thematic analysis approach was used for the whole process [41]. MAXQDA software supported management of the data analysis process. The data analysis consists in six phases: (1) acknowledging the data, (2) creating original codes, (3) finding themes, (4) reassessing the preceding, (5) designating and describing themes, (6) results production. To begin with step (1), group sessions with five project members was organized first to read and coded eight interview transcripts. Thereafter, ensuing step [2], the team generated memos and codes obtaining a set of codes and sub-codes for future analysis. Then, FP, TW, and HM coded the remaining transcripts on their own. This team (FP, TW, HM) had different sessions to discuss key elements and interesting topics and themes, following steps (3), (4) and (5). They also discussed the overall findings from the project using results of all participants and comparing them. This is step (5). Specific for this paper, further and more detailed thematic analysis (step 5 and 6) was conduct by FP to present data on loneliness, social isolation and social capital in the results section of this paper. This meant, re-analysis of all data segment coded using a theme called “Isolation”, and subthemes of the Social Interactions named “[Social Interactions] With Other Prisoners” or “[Social Interactions] With Staff”. All authors reviewed the results section and agreed to the results presented in this paper and interpretation.

## Results

In this section, we present our analysis related to the themes of isolation, social capital, and loneliness, which were not explicitly questioned. These topics were spontaneously reported by participants. Also, in the following, we detail how older incarcerated persons and forensic patients described isolation as an experience that is ubiquitous in detention institutions. Interviewees, mostly if imprisoned, expressed that lack of trust in secure organization play important roles in reinforcing loss of connectedness, increasing social isolation, and diminishing social capital. They also referred to distrust between persons, especially in prisons, and stigma related to mental health as contributing to growing isolation. Although isolation is described as frequent, some imprisoned individuals and patient on forensic units also reported to be able to find connectedness, break isolation, and even make friends regaining social capital.

In the ensuing sections, the first letter of participant’s code (either P or U) refers to the type of place where they receive treatment. P stands for *p*rison and U is for forensic *u*nit. The second letter F refers to *F*rench speaking interviewees, the letter D to German (*Deutsch*) speaking participants and E for *E*nglish speaking study partakers.

### Isolation and loneliness in secure institution

#### Friendship - a challenge leading to social isolation and loneliness

According to the individual deprived of liberty, loneliness is frequent in prison. Study participants often mentioned that they have no contact with other or very few, thus being isolated and feeling lonely. No patient on a forensic unit mentioned this aspect clearly during the interviews, only imprisoned participants did. As stated by one imprisoned participant, « It’s REALLY, REALLY, REALLY, complicated » (PF287) to make friends in prison, even though it was not like that for him before entering prison. Also, proximity is not experienced as necessarily leading to a meaningful relationship between people that are close to one another. Some incarcerated individuals said they shut down when facing others to avoid listening to their problems.

Yes, because you sit very close to each other, you just get … everyone is complaining and although it really doesn’t interest you and because you have to carry your own burden, so that are just things where it can rattle in, where you have to find a way to deal with it. Define yourself, isolate yourself, just close your ears and you have to be able to do that first. (PD268).

It’s not a close contact. I get along with people here, more with some. Of course, sympathy plays a big role. You can get along with one and not with others. It’s just like that. But now what could I say that I have friendships in here? No. I don’t have that either. Maybe colleagues that I work with [in prison]. This I can say. Though, can I actually call someone in here a “friend” or mention “friendship”? No. (PD263).

Hence, forming friendship appears to be a difficult task for these participants. At the same time, the tendency of isolating oneself can even be the incarcerated person’s decision in light of cultural and linguistic differences. Further underlining this point on choosing to remain isolated, a few participants revealed that they wish for some contact, but were not necessarily willing to have many.

No, no, no. As I said before, I spend the morning watching TV and then the afternoon in the cell. The contact I have is while playing cards on Saturday, Sunday and then nothing else. (PF250)

There are days when I really look for and find contact, because the others are opened people like me. But there are days when I don't really want to be in contact with others. I prefer to withdraw a bit and I simply need no one. (PD265)

I would say it’s probably three things. It [not having contact with others] is partially a personal choice, the language and that they are not that interesting to be perfectly honest. [ … ] You know. Persons in prison are not by reputation the most intelligent people on the world and they don't really have that much interesting thing to talk about. (PE252)

Moreover, some participants stated that it is not easy to find someone who understands you and can relate to your personal experience. Age, personal interests, food taste, religious believes and time spent together also play an important role in bonding as outside of prison.

But with my fellow prisoners, it is relatively difficult, considering that we are a group of people thrown together with different characters and sometimes extremely different perspectives. Why are people here so all over the place? It is not really easy to say, “yes, I have really found a comrade”. Now even if you really find a comrade, a colleague or even a friend with whom you can really talk. You can discuss things. You are really interested in discussing this and that with that person. Though it is rather difficult to find someone with whom you can really talk. After the life outside of prison, a friendship like that. It is not easy to find. (PD248)

[ … ] there is the problem of the language, when I think that in [French speaking canton A], for example. For a long time, I was the only Swiss on the whole floor, the oldest. (4 seconds pause) and the only one who spoke French. [ … ] here there are a lot of foreigners. So, I have no contact. It's not possible. [ … ] First, precisely because of the languages, the language. Nationality, the languages, and the interests. (PF286)

#### Institutional rules and organisation worsen social isolation and loneliness

Many interviewees described the worsening of isolation within the walls of their respective institutions (prisons, forensic clinics) as a secondary negative effect due to the fact that these institutions are made to punish and isolate convicted individuals from society. Many sentenced interviewees expressed that incarceration creates poor social capital. Loss of connectedness, lack of trust and aggravation of social isolation are perceived as being caused by restriction of movement between floors, mixing people who do not speak the same language or hindering intimate relationships according to our participants, then leading to loneliness.

The fourth floor is closed in the afternoon from half past one to five thirty for the first month. We don’t have that on the third floor. It seems a bit stupid to me. But otherwise, in general, you should actually be able to include a little bit more people from level four and five in the group activities. Such as tournaments and stuff like that. They are only allowed to do that from level six. That seems a bit stupid to me, because they could also live a little more with the others, or the events in the group, couldn’t they? They would acclimatize much better to the other (PD242)

In the beginning, I was all alone. So, everything was done to prevent me from being in contact with anyone. Not once, have they [prison administrators] put me together with people speaking a language that I can speak. Just so that I had no one to be in contact with. I don't know Romanian, Polish … (PD273)

An experience shared by most interviewees is that separations between friends happen often in prison, thus leading to loss of social capital. They are described as either mandatory or circumstantial. For instance, group changes are mentioned that are mandatory on incarcerated individuals. Moreover, according to the interviewees, even if amities are forged, friends are separated when one of them is released.

These separations are perceived as being difficult for some interviewees who had trustworthy friends.

Then of course I have had a very, very, very good contact with an inmate here. We have had cells next to each other for two years. Our relationship has grown up just like a sisterly relationship. … So, I can talk to her about everything, no matter what it is. Well, we were together in the same living group … Unfortunately, we were separated this year in March this year, because after a certain time you have to change the living group, here in the prison. (PD268)

So, hey, I supported him [an ex-convict]. I wrote to him. So, we had a lot of interactions before because our rooms were in the same corridor, in the common room. Now, well, we communicate via letters. But well, it's all controlled by [prison] services here. Though we stay in touch, because I tell myself, “If I drop him, he has no one behind!” Since, it now seems that his family wants to get back in touch with him, which is a good thing. But if that family doesn't get in touch, he's all alone! [ …]. (PF294)

Loneliness and lack of family contact appears in the preceding briefly when this participant mentioned another incarcerated adult’s experience of prison. Friendships are also depending of time of release.

Of course, depending on the sentence conditions (often not the same punishments [as mine]) it can be that he [the other incarcerated person] gets out earlier or later than me. That [friendship] also depends a lot on these. Then of course it depends on where you are. There are prisons where you have more opportunities to find each other. Here, it is relatively difficult, because there are different floors. I can’t just go to the other floor or something. I also cannot say that someone should come to the same floor as me to have a little more time or something if we get along well. It’s not possible. I just can't. So, it's a little difficult. (PD248)

The possibility to have an intimate heterosexual partner is impossible for this participant on a forensic unit. Isolation is grueling for him as well and appears to be leading to loneliness and loss of social capital.

This is psychological torture, this vengeance of the justice system which makes us completely isolated from life. We are in a ghetto, in a concentration camp without barriers and to some extend it's even worse [ … ] No more relationships with society, no more love relationships with the opposite sex, with women, no more of these. No more meetings of people who could enrich us. There is no more of all that. (UF285)

According to our participant there are only few unsupervised interactions or at least, too little for them. Facilities being unisex, and no heterosexual intimacies allowed (at least according to our results) leads to more isolation and loneliness.

#### Interaction with personnel and possible ways to reduce social isolation and lonelines

Many interviewees reported that contacts with personnel can be very limited for them. Although, they still are interacting on an almost daily basis, some interviewees expressed how bad experiences with staff members diminish sense of trust toward the latter and the fact that they have the impression that the personnel is lacking compassion. This then leads to less trust by individuals deprived of liberty. This inevitably also means that they have less individuals to talk to, leading in turn to more isolation, loneliness and poor social capital.

[ … ] with the officials or with the staff, I have very little contact, because the contact options are actually limited. For example, they are not with us in the cells or corridors. When the door is closed, they simply check. Then, the doors are locked, and the officers go outside in their area. So, they are not in contact with us in principle. (PD248)

It is also related to respect for the staff, but we also have staff in here who I find like, “eh you, the prisoner. Second class or stamp”. “Well zeros”. We also have that. I was also wondering why they don't just quit. What if we're so impossible and it's not fun to work with us? Quit then. Yes. (PD273)

The incarcerated persons we interviewed reported that they do not have meaningful contacts with wards but have more interactions with nurses to reduce their isolation and have more social connectedness, thus better social capital. Interviewees described that nurses evoke mixed reactions and feelings among them. Some interviewees consider nurses as friends, as this one on a forensic unit, “Yes also, two or three good nurses were friends. Yes” (UD241).

Well let's say: It's true that a nurse did help me a lot when my mom died. Yes. I had great difficulty accepting her death in the beginning, but he came to me to show his respect. When he left, he put his hand on my shoulder and said, "Hold on!". You know…. And then this little gesture on the shoulder. It made me feel good! (UF281)

Similar and different circumstances were experienced by older adults in prison. Some find nurses and medical staff important for their social connectedness and personal growth, whilst other do not.

Look, I have a nurse [name] who is very nice to me, who listens to me, et cetera. But the medical staff is mostly nice. There are others that with time you get to know and understand how they work. But normally, I can't say that they're mean or anything. They're nice. (PF274)

Really, I do not see that the people [nurses] with whom I spent time in prison KK were helpful for me. They were not helpful to get me through the particular stage [challenges] I was in. All of it [challenging phase] was extremely emotional. That was probably … It has much to do with the nursing staff (PE252)

### Distrust and loss of social capital

As mentioned in our introduction, trust has been shown to partake in social capital to reduce loneliness and social isolation [22]. Participants depict how the distrust that they experience taints most relationships in prison. Interviewees are worried about the lack of trust especially as most recognize its importance in building social capital in prison. Interviewees reported that they do not build significant relationship with other incarcerated persons because of frequent distrust. Some of them mentioned that they do not talk about mental health issues or their private life with others. Whilst others feared that talking about mental health would stigmatize them and further isolate them from others. To consult a psychiatrist meant to be “crazy” for this participant (PF287). Also, talking about their problems was associated with showing one’s weakness or to reveal secrets put someone at risk for retaliation.

               Interviewer: Do you talk to each other about therapies or your problems?

Participant: No, no. More about politics and things that are in the newspapers or blaming the [name institution in German-speaking Switzerland] a little (both laugh). (PD242)

Well when I got here, they asked me why I'm here, and then I said: “Listen. Sorry, but I don't talk about my private life anymore.” And it stayed that way. When I went to that floor too. Without ever asking me. And finally, they say, "ah he spoke at work, is it true that you did that, that, that?". I said "no, it's not true, because I'm going to judgment again", and that’s it. Because I said, “I don't talk anymore.” I don't have to tell anyone about my private life. (PF250)

Yes, but they asked me several times: “Why are you going to a psychiatrist?” I said, “I’m not feeling well now, like this.” But I didn’t say that I need the psychiatrist and why. I didn’t say that he helps me, and I need him […] No, no, no, no. But if they see here that you are not stable. And I always try to stay strong when I’m with other people…. (PD240).

No, we don’t talk! No, I don’t know. You talk. The penal affaire. The therapy, and all. All of that (It isn’t our business?) and we’re not talking about problems. There are some … Do not speak, do not speak. (PF250).

Although entrusting one another with personal details and mental health issues was described as infrequent in prisons, trusting other imprisoned fellows was perceived as essential in certain situations. One participant mentioned how trust between persons in prisons is important allowing good sharing of living space (PD242). That fact that the prison administration allowed them to use knives (when working in the kitchen) (PD242) was perceived by this participant as a positive experience indicating that the administration trusted them and that they would be valued as human beings similar to the ‘outside world’.

### Positive social interactions to gain social capital

A trusting therapist-patient relationship is essential for most because they have no one else to be close to. This helps reduce isolation further as exemplified by the following: “He/she [the therapist] is the only person in this establishment with whom I can truly open up, because there is no one else. You know (PF249)”. Also, interviewees seemed to agree that friendship and quality relationships (outside of therapy) are almost impossible behind the bars. In some cases, participants reported positive interactions with others. They described their need for trusting others to share their personal experiences and develop meaningful relationships.

I already have most of the contacts with my fellow incarcerated persons or with the bosses at my job [in prison] obviously because most of the time we are with them and they know us a bit better, of course, because we are seven every day, eight hours with them. There is sometimes a way that you can briefly discuss or even hint at a few personal problems. It also depends a bit on the length of time, where you are then. If you work in the same place for two or 3 years, it’s clear, just like outside prison, you would have a little more contact with some people or not. That’s the same. (PD248).

Patients on forensic units seem to have a better living experience than their counterparts in prison.

Yes of course. Yes, I have friends here. Yes, I mean. I still have friends that I speak with. I have a friend. Mr. [name of a third person] who I also speak with. Well, I have another friend who came to see me also it is Mr., hum, [name of a third person] and then another one too. (UF283).

Yes. I have one, who I am very close to. We often drink coffee together. We discuss. Then, there’s another one who comes with me to the therapy. We are also close, but it’s different. It’s different because we’re together less often. Well we see each often, every day, but he likes to stay home. So, do I. We see each other anyway. (UF290).

## Discussion

This study is, to our knowledge, the first to provide qualitative details on the experiences of incarcerated older persons and forensic patients in Switzerland concerning loneliness. Most studies addressed the health consequences of social isolation, loss of connectedness, and loneliness (related to the quality of human interaction). One must remember that loneliness is defined by less interactions with others or of lesser quality leading to unfulfilled social needs [2, 4] as social isolation is the dearth of social relationship in terms of quantity [4, 12, 15]. There are a few studies related to incarcerated adults’ experience in other countries [18, 22, 26, 28, 31, 37]. Nevertheless, our results highlight to which extent the interviewees reported experiencing loneliness, isolation, and distrust in prison and forensic institution leading to reduced social capital in Switzerland and add to the currency body of literature. Social capital is based on emotional or instrumental resources acquired through shared norms and values within durable relationships and is cognitive, like trust, or in relation with networks [22]. Participants to our study also described that friendship and meaningful relationship are possible but rare. These are ways to gain social capital and reduce isolation (quantity of interaction) and loneliness (quality of relationships). Our population is interestingly from two types of institution. Only, one participant in a forensic institution mentioned that rules led him to be more isolated. Most participants that were in this type of institution felt that they were able to make friends and have meaningful relationship with the staff. In general, interviewees that are in prison had a much more critical perspective on their detention center. They more often mentioned the negative aspects leading to isolation. Such factors hinder and reduce possibility of having meaningful friendships by making frequent change and reducing connectedness between incarcerated persons.

Loneliness has been indirectly reported as frequent and had many causes in prison. This was less often mentioned by participants in forensic institutions. Either incarcerated individuals do not wish to be in contact with others (often due to distrust) and isolate themselves, leading to loneliness, or others do not match their personal, religious, or conversation topic preferences. In the latter case, social isolation then is a choice. A main problem seems to be that making friends in prison is not always easy. Moreover, friendships can end abruptly once an incarcerated person is freed, as friends in prison might not be released simultaneously. Making friends is a personal endeavor, which, according to the interviewees, is not supported by detention. Indeed, frequent changes in group composition and difficulty in maintaining contact with outside world lead to reduced connectedness or social capital [4, 18, 26, 28, 42]. A solution was proposed by Archuleta, Prost, and Golder, ([22], p. 30) who note that “these relationships might be facilitated by removing or lessening the restrictions related to visitation or provided greater access to phone contact that does not add burden to older adults who were incarcerated or their family and friends”.

Imprisoned persons have to trust other people so that the information they share is not used against them. Yet, some individuals deprived of liberty are able to form bonds with others and create friendships that last. This was particularly true for our participants in forensic institution. In comparison, prison seems less facilitating with regards to friendship.

As connectedness reduces depression and chronic health conditions among older incarcerated persons [22], we hope that our study underscores the need to facilitate friendships building in secure institutions despite the challenges these environment and general distrust pose.

As experienced by the general population, isolation and loneliness can worsen mental health and increase mortality [2, 15, 18, 19, 43]. Worsening of mental health has been shown to aggravate feeling of loneliness and social isolation in the older person [19, 43] and to increase risk of suicide [30, 31]. Rapid cognitive decline was observed in relation with isolation and loneliness worldwide in longitudinal studies [20, 21]. A systematic review by Boss, Kang, & Branson (2015) [44] showed a similar tendency.

Our results show that prison and forensic organization could implement changes to reduce structural limitations that hinder social connectedness, hence reducing isolation and loneliness. Based on the principle of equivalence of care it is critical to optimize factors that improve mental health and thus allow friendships to flourish in prison. Reducing unnecessary changes in division of older imprisoned individuals could be an easy means to this end. Working and living groups should be maintained actively as long as possible. Prison administrations should try to improve connectedness with family members from outside prison with phone calls and visits as mentioned by incarcerated interviewees, and recent literature [22]. Friendship continuity after liberation should also be promoted both in prison and forensic institution.

Measures facilitating social connectedness will help persons deprived of their liberty to gain more social capital, that is, resources such as emotional (group psychotherapy, team sport) or instrumental support for genuine relationships (phone calls, emails, family meetings) both inside and outside of detention in prison or forensic institution. Raising social capital will be done by reducing stigma related to mental health, encouraging trustworthy relationships or making better network structure [22, 24, 25]. Trust is the basis of good human relationships. In secure institutions, trust is often fragile. As interaction with staff is often transactional or related to punishment, study participants that were in prison considered them mostly as punishers and order-keepers. This creates lack of trust and incarcerated persons do not build positive relationships with wards. All these factors are described as creating more isolation for the participants in prison.

Participants in this study highlighted the important role of health care personnel. While healthy human relationships between incarcerated persons are hindered by distrust, some interviewees stated that nurses and therapists are trust-worthy especially on forensic units. They mentioned spontaneously how nurses and therapists reduce isolation and help connectedness. This is also true in prison but was not mentioned as often by our participants. These healthcare professionals can thus not only provide incarcerated people with mental health support and care, but they seem also to become a replacement for the lack of other positive social contacts. It relays the value of therapeutic relationship that are not enough recognized in this context.

Interviewees also described their positive experiences resulting from the fact that they are allowed to use knives when they work in the kitchen. They feel valued by the prison administration and others working with them because they feel that they can trust each other with sharp objects. This has been mentioned in previous literature as well [23, 45].

Additionally, stigmatization surrounding mental health problems has been described as reducing the imprisoned person’s will to open up to others, i.e. to trust them [26, 27]. This was also shown in our interviews. Relationships marked by stigma are also lacking closeness and intimacy [4]. Stigma associated with mental disease limits contact of older incarcerated persons with others as they feel inappropriate and unworthy of social relationships. Hence, the social inadequacy, which they feel as being insufficient or even laughable, leads to mistrust [18, 26, 42]. Being in the margin also creates lack of connectedness [18]. Our results underline the need to put in place measures to reduce stigmatization of mentally ill persons in prison and forensic institution to help them gain social capital. Indeed, trust in others is an essential aspect of social capital. The latter can reduce both physical and mental health problems. The need to be helpful to younger generation is also valuable for older persons deprived of liberty, even more so in prison [46]. Elders are usually more experienced and can help their younger conterparts to cope with imprisonment, mental health problems or measures. This might constitute a way to cope with isolation in secure institution as well. Many interventions were proposed to reduce social isolation and loneliness in literature for the older person [3, 17, 47]. They have to be individualized as they are experienced differently by the older person [3]. Hence, stakeholders in prison and forensic contexts should inspire their intervention based on the recent literature.

### Limitations

Our study has common limitations associated with qualitative study designs. Social acceptability might have limited individuals to speak their mind. They might have chosen the answer that one is expected to give in certain situation, in a certain culture. Also, a volunteering bias is possibly present. Isolated individuals might have felt the need to break isolation by participating in such a study but informing us on this pressing issue even more accurately. Thus, our findings do not represent the experience of every person deprived of liberty in prison or on a forensic unit. Most importantly, as evident from the interview guide information presented in the methods section, participants were not specifically asked about loneliness and isolation in prison. These data generally came out from the discussions on general life in prison and being an older person. Thus, data presented can be incomplete and we do not claim that our results are generalizable to other contexts. We have participants in prison or on forensic unit and most of the results related to isolation, poor social capital and connectedness are related to imprisoned persons. Positive experiences are related most of the time to the forensic unit. Our project, as it is exploratory, did not have the power to draw frank comparison. However, this is an interesting difference that should be investigated in future research. Also, this heterogeneity highlights that confinement to an institution causes isolation – no matter in what specific setting it takes place. Since the different settings and treatment options might have differing impacts on the feelings of loneliness, this should be explained in future research.

## Conclusion

We brought to light the fact that loneliness, social isolation, and lost connectedness are frequent in secure institutions (prison and forensic unit). As the literature on those topics showed, they all linked to worsening of mental and physical health. In turn, poor mental health leads to a worsening feeling of loneliness and social isolation [10, 19, 43]. These need to be addressed by allowing imprisoned older adults to gain social capital, which can be achieved by increasing connectedness and trusting relationships. Those are essential to humans [43]. Prison can become a health-promoting environment through connectedness, friendship, and trust promotion. It is a matter of public health [19]. Improving social capital is essential “to tackle social inequalities that undermine mental health in marginalised groups” ([48], p. 6) improve health of older adults [49]. Changes will have to be made by stakeholders to achieve this goal, such as facilitating friendship between incarcerated persons, allowing more interaction with the outside, and educating prison personnel on how to gain imprisoned individuals trust, and reduce stigma. Understandably, more research will need to be done to assure its well-balanced implementation, and to better understand barriers and enablers to do so.

## Supplementary Information

**Additional file 1.**

## Data Availability

Our dataset is not publicly available due to confidentiality concerns. As, our analysis is based on qualitative interviews with older incarcerated persons and their privacy could be compromised if we shared the whole transcripts publicly. Our first goal is to protect those vulnerable participants’ privacy. Nevertheless, upon reasonable request, we are willing to share our transcripts. The point of contact to access this data and material is Mrs. Anne-Christine Loschingg at the Institute for Biomedical Ethics at the University of Basel (a.loschnigg@unibas.ch).

## Abbreviations

COVID 19: Coronavirus disease 2019
UK: United Kingdom
COREG-32: Consolidated criteria for reporting qualitative research: a 32-item checklist for interviews and focus groups
