# Supplementary material for: Loneliness and social isolation among the older person in a Swiss secure institution: a qualitative study
Source: BMC Geriatr. 2022 Feb 1;22:90. doi: 10.1186/s12877-022-02764-7 (PMC8806358; doi:10.1186/s12877-022-02764-7)
Supplement: Supplementary file 1 — Additional file 1. [file 12877_2022_2764_MOESM1_ESM.pdf]

Interview Guide for Imprisoned Adults

For the article entitled:

"Loneliness and Social Isolation among the Older Person in a Swiss Secure Institution: A qualitative study"

Authors and order:

Felix Pageau[1], Helene Merkt[1], Elmar Habermeyer[2], Bernice Elger[1,3], Tenzin Wangmo[1]

[1] Institute for Biomedical Ethics, University Basel, Switzerland.

[2] Psychiatrische Universitätsklinik, Klinik für Forensische Psychiatrie, Zurich, Switzerland.

[3] Center for legal medicine, University of Geneva, Switzerland.

To:

BMC-Geriatrics Editorial Board

January 4th, 2021

**Interview questions for imprisoned adults**
Could you describe a typical day here in [name of the institution]?
Are you close to anybody within the institution?<br>• Staff<br>• other inmates<br>Do you keep in touch with anybody outside of the institution?
What do you think about the mental health care services in [name of the institution]?
What kind of mental health disorders do you have?<br>• How long have you had this disorder for?<br>• Since when have you received treatment for your disorder?
When did you first get in touch with the mental health care service?<br>• How?<br>• Who?<br>• Why?
What **type** of treatments do you receive for your mental disorder?<br>• In the institution<br>• Before imprisonment<br>• Any differences?<br>• Frequency - how often?

• Which specialists did you meet?<br>• Duration of a session?
Do you think that anything has **changed** about your mental health while being here?<br><br>• Do you feel any improvement or degradation about your mental health issues?<br>• What treatment helps/has helped you most?<br>• Are you satisfied with these treatments?<br>• What treatment/activity would you most preferably drop?<br>• Do you think that you're treated differently because of your age?
• Do you talk to anybody of the other inmates about being in touch with mental health services?<br>• Has anything changed since you contacted the mental health service/you entered the institution?
If you could change anything about the treatment you receive, what would it be?<br><br>What type of additional health care would you need?
**Elicitation Technique**<br><br>Where do you place the mental health professional?

Where do you place the person conducting the forensic psychiatric expertise?
What do you do yourself about the mental health issues that you have?<br><br>What influences the **progress** of your therapy?<br><br>Who influences the progress of your therapy?
What is it like to be here? What is it like to become older here?<br><br>What is **most challenging concern** for you in your life right now?<br><br>• Over your course of the stay in your institution, what has changed for you? Do you face different challenges now?<br>• Do you think that aging has an impact on your everyday life in the institution?<br><br>Is the **prison environment** adapted to people of your age and older?<br><br>• What would you improve or need?
What **plans** do you have with your life?

How would you advise a younger inmate who asks you how to deal with mental illness?
